# Changes in Patient-Reported Health Status in Advanced Cancer Patients from a Symptom Management Clinic: A Longitudinal Study Conducted in China

**DOI:** 10.1155/2022/7531545

**Published:** 2022-09-16

**Authors:** Yening Zhang, Zimeng Li, Ying Pang, Yi He, Shuangzhi He, Zhongge Su, Yuhe Zhou, Yan Wang, Bingmei Wang, Lili Song, Jinjiang Li, Xinkun Han, Chengcheng Zhou, Xiumin Li, Lili Tang

**Affiliations:** Department of Psycho-Oncology, Key Laboratory of Carcinogenesis and Translational Research (Ministry of Education/Beijing), Peking University Cancer Hospital & Institute, Beijing, China

## Abstract

**Objectives:**

The integration of patient-reported health status has been increasingly emphasised for delivering high-quality care to advanced cancer patients. This research is designed to track health status changes over time in Chinese advanced cancer patients to explore the risk factors affecting their health status.

**Methods:**

Advanced cancer patients were recruited from Peking University Cancer Hospital. An electronic patient-reported outcome (ePRO) system with validated measurements was used to collect the data. ANOVA, the chi-square test, the nonparametric Kruskal–Wallis H test, and generalized estimating equation (GEE) analysis were used for the data analysis.

**Results:**

One hundred and three patients completed a baseline survey (*T* = 0) and two follow-up surveys (*T*1 = 14 days, *T*2 = 28 days). Chi-square test results indicate a significant decrease in the percentage of patients reporting moderate or severe difficulty experienced by patients in terms of mobility, pain/discomfort, and anxiety/depression. However, there is a significant increase in the percentage of patients reporting moderate or severe difficulty in self-care and usual activities. Scores on the visual analogue scale in the EQ-5D-5L instrument (EQ-VAS) are associated with patients' income, and the degree of moderate or severe anxiety/depression is found to be associated with employment status. The GEE results show that pain, loss of appetite, poor walking status effected by symptoms, depression, and anxiety has worsened the health status.

**Conclusions:**

The health status of Chinese advanced cancer patients under ePRO follow-up in China significantly improves in the physical and psychological dimensions, accompanied by a decrease in usual activities and self-care. Routine screening and rational supportive care are recommended in oncology for cancer care. Based on the rational application of ePRO, longitudinal studies exploring the potential mechanisms of health status changing would provide more beneficial guidance for improving the quality of life in patients with advanced cancer.

## 1. Background

Cancer patients experience subjective distress induced by various symptoms related to both the disease itself and treatment-related adverse events [1, 2]. Though the five-year survival rate for cancer in China has increased from 30.9% recorded in 2003 to 40.5% in 2015 [3], it is still lower than the rates in developed countries [4]. Available data indicate that approximately 60% of cancer will progress to the advanced stages; symptom burden would be complicated and difficult to manage in this population [5, 6]. Quality of life (QoL) or health status improvement is the primary objective of high-quality care for advanced cancer patients. Studies on cancer-related mechanisms, patient-reported outcome (PRO) monitoring, and multidisciplinary interventions for improving QoL in this population have been reported in various research publications. Recurrent or persistent inflammation is a common factor in the pathogenesis of neoplasia [7], and QoL is associated with systemic inflammation in patients with advanced cancer (based on PRO measurements) [8]. Some supportive interventions have been proven to improve QoL by modulating inflammatory mediators [9, 10]. PRO monitoring of subjective symptoms in advanced cancer patients will provide a detailed map of health status changes and is recommended in the clinical practice guidelines on palliative care of the National Comprehensive Cancer Network (NCCN) [11]. PRO that includes health status is also viewed as a crucial indicator of treatment effectiveness in clinical trials and is an essential criterion for drug approval, as required by the U.S Food and Drug Administration (FDA) from 2006 [12]. However, both physical and psychosocial symptoms can adversely affect QoL and daily functions [13]. Research shows that improvement in the QoL of patients is of great significance for their anticancer treatments, long-term rehabilitation, and survival. Kypriotakis et al. indicated that advanced cancer patients' longitudinal experience of QoL is a significant prognostic factor for survival [14].

Describing the changing trends in the health status, exploring the mechanisms behind fluctuating symptoms, and designing efficient interventions for advanced cancer patients would be particularly beneficial for improving QoL of this population. There have been several longitudinal studies focused on cancer patients. Van Dijk-Lokkart et al. reported that cancer-related fatigue can improve after treatment in children diagnosed with cancer, which is a favourable prognosis for a subsequent increase in physical activity [15]. For cancer patients undergoing pelvic/abdominal radiotherapy, effectively managing nausea resulted in improved sleep [16]. Findings from a follow-up study indicated that QoL became worse for head and neck cancer (HNC) patients after cancer treatment [17]. Another study reports that the QoL of early-stage patients with non-small cell lung cancer deteriorated six weeks after video-assisted thoracoscopic lung resection and had improved by 12 months after the surgery [18]. A few of these studies focus specifically on changes in QoL in advanced cancer patients. The study by Contogni et al. focuses on changes in QoL in advanced cancer patients on parenteral nutrition (HPN) [19]. Deteriorating nutrition status would negatively influence QoL in advanced colorectal cancer [20]. Based on a longitudinal study, Rojas-Concha suggests that the QoL of advanced cancer patients in Chile could benefit from palliative care [21]. Research on the longitudinal health status of patients with advanced cancer is of particularly crucial clinical significance, especially for those who no longer have the opportunity for effective anticancer treatments.

We conducted this study based on a hypothesis drawn from research studies by Bash et al., which has indicated that PRO symptom monitoring is beneficial for the QoL and eventual survival of advanced cancer patients [22, 23]. Hence, we are conducting a pilot study to assess whether significant improvements in QoL can be tracked in Chinese advanced cancer patients using PRO monitoring. There are still a few knowledge gaps in the literature on this area of research in China. First, previous national studies are either on a single cancer type or have cancer survivors as participants and hence cannot be generalized to advanced cancer patients whose cancers are unlikely to be cured or controlled with anticancer treatments. Second, the extant research does not include longitudinal studies and none of the studies monitor changes in the health status of advanced cancer patients. Therefore, the objectives of this study are as follows: (1) to track changes in health status over time in Chinese advanced cancer patients registered on multiple ePRO QoL assessment platforms, (2) to examine discrepancies in the EQ-5D-5L results in patients with different demographic and medical condition, and (3) to explore risk factors that influence the changing of health status in advanced cancer patients.

## 2. Materials and Methods

This longitudinal study was conducted at Peking University Cancer Hospital. Patients who visited the symptom management clinic at Peking University Cancer Hospital between June 1st to December 31st, 2019 were recruited as participants in the study on their initial visit. The inclusion criteria were as follows: (1) aged ≥ 18 years old; (2) diagnosis of an advanced cancer (UICC TNM classification stage III without curative treatment chance and stage IV), including lung cancer, gastric cancer, oesophageal cancer, liver cancer, colorectal cancer, and breast cancer; (3) able to sign informed consent; and (4) could understand the items. Patients were excluded if they had a history of severe mental disorders or major communication difficulties. The study was approved by the Institutional Research Board (IRB) of Peking University Cancer Hospital (approval number 2019YJZ07).

### 2.1. Measures

EQ-5D-5L. The EQ-5D-5L instrument comprises a short descriptive system questionnaire and a visual analogue scale (EQ-VAS) [24]. Each respondent is asked to choose a digital number that best describes their health status for the day on each of the five dimensions, and the response for each health status dimension is assigned a five-digit code. The EQ-5D-5L health status results are then converted into a single index value for China [25]. The EQ VAS records the self-rated overall health status of the respondent. The EQ-5D-5L instrument is used to assess the participants' health status on the day of evaluation. The EQ-5D is a preference-based measure of health status and QoL used worldwide in clinical trials, population studies and real-world clinical settings. It was developed from the EQ-5D-3L [24], the EQ-5D-3L was introduced in the 1990s [26], comprises five dimensions: mobility (MO), self-care (SC), usual activities (UA), pain/discomfort (PD), and anxiety/depression (AD) and has three levels in each dimension: no problems, some problems, and extreme problems. Although widely used in the clinical trials, the EQ-5D-3L instrument also has several limitations; for example, it is not sensitive to mild health changes and it suffers from ceiling effects [27]. To solve the issues, a new five-level EQ-5D (EQ-5D-5L) instrument was developed by the EuroQol Group. It remains the original dimension and expands the number of levels of severity in each dimension from three to five: no problems, slight problems, moderate problems, severe problems, and unable to/extreme problems; thus defining 3,125(55) distinct health statuses. The EQ VAS records the self-rated health status valuation of the respondent on a vertical visual analogue scale, with the end points labelled ‘The best health you can imagine' (100 score) and ‘The worst health you can imagine' (0 score). The measurement results of the EQ-5D-5L instrument can be used to generate health utility values using value sets. It is generally suggested that different value sets reflect the health preferences of people in different countries. Currently, many countries, including China, have developed EQ-5D-5L value sets based on the health preferences of their respective populations [25, 28, 29]. We used the EQ-5D-5L tariff suggested by Liu in this study [30].

The Chinese version of the MD Anderson Symptom Inventory (MDASI-C). It is a widely used multisymptom inventory with 19 items (13 items for symptom severity and six items for life interference) rated on a 0–10 scale on which 0 = nothing and 10 = most severe. MDASI-C is used to assess the symptom severity and the degree of life interference over the past 24 hours. As proposed by Cleenland et al., moderate-to-severe symptoms were defined as scores of ≥5 on the MDASI [6]. The MDASI-C has passed reliability and validity tests and can be used to measure the severity of multiple symptoms and their impact on function [31].

The Hospital Anxiety and Depression Scale (HADS). It has 14 items, with a 0–3 score range for each item. It is used to measure the anxiety and depression symptoms of patients over the past two weeks and is a relatively complete assessment with good reliability and validity [32].

The case report form (CRF). It captures the following: (1) demographic and social economic information, such as age, sex, occupation, education, marital status, and medical payments; (2) disease data, such as information on disease diagnosis, staging, treatment, and medication; (3) The Charlson Comorbidity Index (CCI) [33] is used to evaluate complications that have a significant impact on the survival and prognosis of cancer patients.

The Eastern Cooperative Oncology Group Performance Status (ECOG-PS) scale. It is a widely used tool for measuring the current functional status of cancer patients on five levels [34]: 0 = normal with no limitations; 1 = not my normal self, but able to be up and about with fairly normal activities; 2 = not feeling up to most things, but in bed or chair less than half the day; 3 = able to do little activity and spend most of the day in bed or chair; and 4 = pretty much bedridden, rarely out of bed.

### 2.2. Data Collection

All data were collected on Day 0 (T0), Day 14 (T1), and Day 28 (T2) after initial recruitment via an electrical PRO (ePRO) system developed by the researchers, with participants completing the baseline assessment under the guidance of research assistants. Several assessments and training sessions were held for research assistants, and their competence (especially evaluation consistency) was evaluated before commencing the study. The symptom management clinic of Peking University Cancer Hospital requires patients to make a follow-up visit every 14 days. Thus, we designed the follow-up time points of the study as T1 (Day 14) and T2 (Day 28), on which the patients received a reminder message from the ePRO system to complete the follow-up assessment via mobile phone. If the participant had moderate-to-severe physical and psychological symptoms, both the patients and the doctors were immediately sent alerts: reminding the patients to visit the clinic promptly and informing the doctors to make timely adjustments to the patient's medication.

### 2.3. Statistical Analysis

The EQ-5D-5L results were calculated following the user guide by the EuroQol Research Foundation [35]. The frequencies and proportions of the EQ-5D-5L dimensions and levels are presented using descriptive analysis. Moderate-to-severe difficulty was defined as a dimension-level score of ≥3. The overall health status was measured using the EQ VAS, employing mean and standard deviation. The EQ-5D-5L index was calculated using the Chinese value set and presented as the mean and standard deviation values. To explore discrepancies in the EQ-5D-5L results recorded over time against demography and medical data, chi-square tests were used, along with one-way ANOVA (if the homogeneity of variance assumption was satisfied) and the nonparametric Kruskal–Wallis H test (if homogeneity of variance assumption was not satisfied). Generalized estimated equation (GEE) analysis was applied to repeated measures of EQ-5D-5L and to explore the risk factors for changes in health status. All demographic information, medical data, and MDASI-C and HADS data were included in the GEE model. The statistical analyses were all conducted using SPSS 25.0 (IBM Corporation).

## 3. Results

### 3.1. Demography, Medical Data, and Other Descriptive Results at Baseline

**Figure 1:**
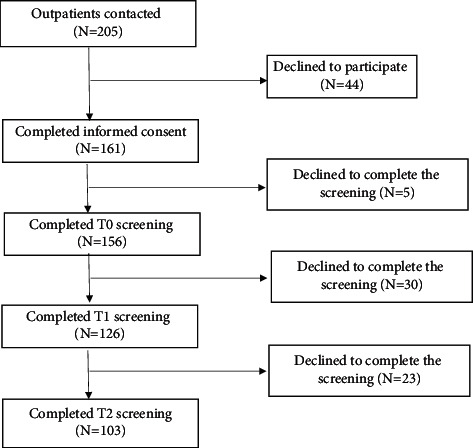
Study flowchart.

One hundred-and-sixty-one advanced cancer patients were recruited as participants and completed informed consent, with 156 participants completing the survey at baseline, 126 participants completing the first two surveys and 103 participants completing all three surveys (flowchart shown in Figure 1). The mean age of our sample was 56.22 ± 10.898, with most of these individuals being middle-aged and elderly (age ≥ 45) (*n* = 125, 55.7%), living with a spouse (*n* = 147, 94.2%), and having their medical costs covered by a government-pay scheme or a medical insurance policy (*n* = 129, 82.7%). Half the patients with cancer have a progress duration of less than half a year. The results of the one-way ANOVA and nonparametric Kruskal–Wallis H test indicate that discrepancies in the EQ-5D-5L dimension responses correspond to specific demographic characteristics. There was a significant discrepancy in the EQ-5D-5L VAS mean scores of patients with different incomes: patients with an income of 15,000 Yuan per month and higher reported poorer health status (measured via VAS scores) than those with lower incomes. Furthermore, there was a significant discrepancy in the Dimension 5 (anxiety/depression) responses of patients with different employment statuses: unemployed patients reported higher levels of anxiety/depression than those employed (Table 1).

The top five symptoms reported as moderate-to-severe by advanced cancer patients are as follows: fatigue (61.5%), insomnia (60.9%), pain (58.3%), distress (53.2%), and loss of appetite (46.8%). 57.1% of the participants reported experiencing significant mood distress via the HADS (score of ≥ 15).

### 3.2. Health Status Measured via EQ-5D-5L at Baseline and Trends at Three Time Points

Significant discrepancy was found via the chi-square test between the three time points, T0 vs. T2 and T1 vs. T2. Responses with moderate and severe difficulty changed significantly in the following dimensions: decreased in mobility (three time points, *χ*^2^ = 84.541; *T*1 vs.*T*2, *χ*^2^ = 60.438; *T*0 vs.*T*2, *χ*^2^ = 55.060; *p* < 0.001), pain/discomfort (*three time points*, *χ*^2^ = 136.303; *T*1 vs.*T*2, *χ*^2^ = 88.542; *T*0 vs.*T*2, *χ*^2^ = 99.651; *p* < 0.001); and anxiety/depression (*three time points*, *χ*^2^ = 91.625; *T*1 vs.*T*2, *χ*^2^ = 60.027; *T*0 vs.*T*2, *χ*^2^ = 64.254; *p* < 0.001) dimensions; but increased in self-care (*three time points*, *χ*^2^ = 50.202; *T*1 vs.*T*2, *χ*^2^ = 33.052; *T*0 vs.*T*2, *χ*^2^ = 29.277; *p* < 0.001), and usual activities (*three time points*, *χ*^2^ = 78.562; *T*1 vs.*T*2, *χ*^2^ = 53.007; *T*0 vs.*T*2, *χ*^2^ = 48.228; *p* < 0.001) dimensions (Table 2). The mean scores of the EQ-5D-5L index and the VAS values at the three time points improved slightly but not significantly (Table 2).

### 3.3. Risk Factors for Health Status from the Longitudinal Study

The results from the GEE model show that ECOG-PS scores (OR = 0.910, *p* < 0.001), pain (OR = 0.984, *p* = 0.005), poor walking status effected by symptoms (OR = 0.972, *p* < 0.001), and anxiety/depression (OR = 0.991, *p* < 0.001) were risk factors, which were strongly associated with changes in the health status of patients with advanced cancer (Table 3).

## 4. Discussion

### 4.1. Main Findings

The EQ-5D-5L has been used extensively to explore health status among different populations. We found that health status scores among advanced cancer patients (VAS score: 58.35, index value: 0.614) were significantly lower than the norm scores of the Chinese population (VAS score: 85.4, index value: 0.932) [36] and those of cancer survivors in general (VAS score: 70.35, index value: 0.841) [35]. Su et al. report that lung cancer patients have the lowest health-related QoL compared to other cancer patients [37]. However, in our study sample, we found no discrepancies between advanced cancer patients with different diagnoses. Several recent studies have shown that the health status is increasingly viewed as a predictor of survival [38]. This was confirmed by Kypriotakis et al. with respect to advanced cancer patients; they concluded that longitudinal experience of health status is a significant prognostic factor for survival and holds important implications for medical decision-making concerning advanced cancer patients [14]. If health status monitoring is indeed beneficial for survival, it would be more adoptable—from the perspective of health economics—than expensive anticancer treatments. Discrepancies in health status were found among patients with different demographics and medical data. Patients with monthly incomes of 15,000 Yuan per month and higher reported poorer health statuses, as reflected by their EQ-5D-5L VAS scores, than those with lower incomes. This finding is similar to one of the findings in the research by Tribius, who reports that locally advanced HNC patients with a high socioeconomic status reported worse QoL than similar patients with a low economic status [39]. Furthermore, a larger proportion of unemployed patients reported moderate problems on the anxiety/depression dimension than patients who were employed. By contrast, Morrison reports that lung cancer patients are more likely to report emotional problems upon diagnosis if they are employed [40]. Discrepancies may also occur because the compared samples are different, which indicates that the nature of the emotional challenges experienced by advanced cancer patients is different from those experienced by other populations.

Symptoms, function and mood can significantly influence the health status among advanced cancer patients. In our study, pain, poor walking status effected by symptoms, and anxiety/depression are risk factors that significantly influence the health status of advanced cancer patients. Pain is a factor influencing poor health status, which calls for more attention to pain management for advanced cancer patients, as indicated by the high prevalence of pain in our sample (58.3%). Walking status effected by symptoms has also been confirmed as another factor for low health status by Laird et al., who reported that, among advanced cancer patients, performance status is strongly associated with deteriorating QoL parameters [8]. Dunn et al. indicated that advanced melanoma patients have more significantly decreased emotional function than patients with a localised form of the disease. A high proportion of patients in our sample reported significant psychological distress, which is an independent risk factor for deteriorating health status. High-quality supportive care for advanced cancer patients should be a key part of the strategies in the dimensions of symptom management, performance status improvement, and psychosocial care.

In this longitudinal research, health status changes were captured via sensitive EQ-5D-5L screening, with results indicating that symptom monitoring is of great significance to rational symptom management. Changes in the health status were captured at three time points, with significant changes occurring between *T*0 and *T*2 and between *T*1 and *T*2, but not between *T*0 and *T*1. This indicates that in advanced cancer patients, significant health changes may occur approximately one month. In this study, all participants utilised the ePRO auto-symptom management system. Symptom alerts, triggered by a score of ≥7 (severe symptom burden) on each symptom scale in the MDASI, would appear in patients' ePRO terminals, prompting these patients to counsel with research coordinators and receive symptom management knowledge, if they so choose. In our study, pain, anxiety and depression can improve with timely adjustments of symptoms management informed by the ePRO system. This is supported by the findings of Basch et al., who concluded that advanced cancer patients receiving symptom monitoring are admitted to the emergency room less often, remain on chemotherapy longer and have a longer quality-adjusted survival rate than those who do not receive symptom monitoring [22]. Furthermore, overall improvement in the survival rate has been confirmed via a clinical trial: 31.2 months among the PRO group vs. 26.0 months among the usual care group [23]. It is necessary to explore whether the benefits would be derived from ePRO system implementation in terms of the health status and survival in Chinese advanced cancer patients.

Many studies have proved that common symptoms with a high prevalence among cancer patients are modulated by inflammation, patients with high C-reactive protein (CRP) levels are at greater odds of experiencing fatigue [41], and significantly high levels of vegetative depression are strongly linked to elevated levels of interleukin-6 (IL-6) [42]. Inflammation is one of the key factors that modulate cancer pain, as proinflammatory cytokines and chemokines modulate neuronal activity. Corticosteroids can relieve pain when administered as anti-inflammatory drugs [43]. Both physical and psychological symptoms associated with inflammation are independent risk factors for fluctuating health status. Furthermore, inflammation negatively impacts cancer prognosis, which is associated with diminished QoL [44]. The results of this research have also inspired us to further investigate the fluctuation of inflammatory mediators in relation to changing in health status among patients with advanced cancer.

### 4.2. Limitations

The limitations of this study are as follows: the sample size is small and the study was conducted at a single centre. A multicenter study with a more representative sample is recommended for future research. This longitudinal study was designed primarily to investigate changes in health status among advanced cancer patients; hence, a comparison group was not concluded. However, we compared our results against those of the normal Chinese population and cancer survivors. The findings of this study indicate that advanced cancer patients could benefit from routine health status monitoring. Therefore, we recommend that a random clinical trial designed specifically to investigate the benefits of the ePRO system to the health status of advanced cancer patients be conducted.

### 4.3. Clinical Implications

For advanced cancer patients, curative care is not the dominant medical strategy. However, this population would benefit more from supportive care that focuses on how to improve the patient's health status. It is necessary to monitor the health status of advanced cancer patients using a validated ePRO platform and to develop individualised supportive care protocols. Pain management, improving mobility, and psychosocial care for anxiety and depression should be incorporated into supportive care protocols.

## 5. Conclusion

Monitoring the health status of advanced cancer patients and developing individualised supportive care protocols are imperative for positive outcomes. The EQ-5D-5L is a useful tool for recording patients' health status via dimension responses, index scores and the VAS, as well as for capturing changes in the health status over time under reasonable symptom management or supportive care. The risk factors for deteriorating health status can serve as useful references for health status management in advanced cancer patients, especially for symptom management.

## Figures and Tables

**Table 1:** Demographic information, medical data, and distribution of EQ-5D-5L dimension responses (% of responses with moderate problems—i.e., Level 3—and above) as a function of various demographic characteristics.

Demographic characteristics	*N* (%)/*M* ± SD (*N* = 156)	Mobility *N* (%)	Self-care*N* (%)	Usual activities *N* (%)	Pain/discomfort *N* (%)	Anxiety/Depression *N* (%)	EQ-5D-5L index *M* (SD)	VAS *M* (SD)
*Age (years)*	56.22 ± 10.898							
≤44	23 (14.7)	6 (26.1)	3 (13.0)	6 (26.1)	9 (39.1)	10 (43.5)	0.67 (0.33)	61.78 (24.34)
45–59	64 (41.0)	20 (31.3)	6 (9.4)	17 (26.6)	43 (67.2)	26 (40.6)	0.59 (0.31)	56.58 (23.36)
≥60	61 (39.1)	20 (32.8)	9 (14.8)	20 (32.8)	35 (57.4)	17 (27.9)	0.63 (0.31)	59.10 (21.00)
Missing	8 (5.1)							
*P* value		0.355	0.616	0.818	0.060	0.072	0.606	0.610
*Sex*								
Male	86 (55.1)	23 (26.7)	8 (9.3)	21 (24.4)	51 (59.3)	28 (32.6)	0.65 (0.29)	60.26 (20.45)
Female	69 (44.2)	26 (36.2)	12 (17.4)	24 (34.8)	41 (59.4)	29 (42.0)	0.58 (0.35)	55.48 (23.91)
Missing	1 (0.6)							
*P* value		0.529	0.815	0.193	0.527	0.148	0.378	0.263
*Marital status*								
Without partner (single, separated, divorced, or widowed)	8 (5.1)	44 (29.9)	19 (12.9)	42 (28.6)	88 (59.9)	55 (37.4)	0.61 (0.32)	57.98 (22.41)
With spouse	147 (94.2)	4 (50.0)	1 (12.5)	3 (37.5)	4 (50.0)	2 (25.0)	0.61 (0.37)	60.88 (16.39)
Missing	1 (0.6)							
*P* value		0.233	0.972	0.589	0.581	0.480	0.737	0.742
*Education level*								
Junior, middle school, and lower	49 (31.4)	12 (24.5)	6 (12.2)	11 (22.4)	31 (63.3)	17 (34.7)	0.63 (0.30)	57.57 (23.08)
High middle school and special secondary school	45 (28.8)	15 (33.3)	7 (15.6)	17 (37.8)	26 (57.8)	18 (40.0)	0.59 (0.35)	56.16 (23.91)
Junior college and above	61 (39.1)	21 (34.4)	7 (11.5)	17 (27.9)	35 (57.4)	22 (36.1)	0.63 (0.31)	60.03 (20.01)
Missing	1 (0.6)							
*P* value		0.494	0.815	0.256	0.797	0.859	0.794	0.659
*Average family income*								
<5,000 Yuan/month	42 (26.9)	13 (31.0)	5 (11.9)	14 (33.3)	26 (61.9)	19 (45.2)	0.60 (0.30)	58.67 (20.31)
5,000–10,000 Yuan/month	50 (32.1)	13 (26.0)	7 (14.0)	12 (24.0)	29 (58.0)	15 (30.0)	0.65 (0.33)	60.36 (22.10)
10,000–15,000 Yuan/month	40 (25.6)	14 (35.0)	4 (10.0)	11 (27.5)	22 (55.0)	11 (27.5)	0.63 (0.30)	61.55 (21.66)
15000 Yuan/month and above	23 (14.7)	8 (34.8)	4 (17.4)	8 (34.8)	15 (65.2)	12 (52.2)	0.53 (0.37)	46.35 (23.56)
Missing	1 (0.6)							
*P* value		0.794	0.850	0.706	0.852	0.108	0.516	*p*=0.044^*∗*^
*Medical cost coverage*								
Government-pay/Medical insurance	129 (82.7)	40 (31.0)	15 (11.6)	38 (29.5)	74 (57.4)	45 (34.9)	0.63 (0.31)	59.17 (22.74)
Self-pay	26 (16.7)	8 (30.8)	5 (19.2)	7 (26.9)	18 (69.2)	12 (46.2)	0.55 (0.34)	52.96 (18.17)
Missing	1 (0.6)							
*P* value		0.981	0.293	0.796	0.263	0.278	0.161	0.159
*Employment status*								
Retired	54 (34.6)	17 (34.5)	9 (16.7)	19 (35.2)	33 (61.1)	19 (35.2)	0.60 (0.34)	59.46 (22.39)
Employed	71 (45.5)	22 (31.0)	5 (7.0)	16 (22.5)	38 (53.5)	20 (28.2)	0.66 (0.28)	60.20 (22.49)
Without work	28 (17.9)	9 (32.1)	5 (17.9)	8 (28.6)	20 (71.4)	17 (60.7)	0.52 (0.36)	50.11 (20.03)
Missing	3 (91.9)							
*P* value		0.994	0.172	0.299	0.253	*p*=0.010^*∗*^	0.138	0.107
*Cancer site*								
Breast	18 (11.5)	7 (38.9)	5 (27.8)	8 (44.4)	9 (50.0)	9 (50.0)	0.51 (0.44)	55.28 (28.44)
Gastric	21 (13.5)	2 (10.0)	1 (5.0)	1 (5.0)	7 (35.0)	5 (25.0)	0.78 (0.19)	59.05 (19.15)
Oesophageal	10 (6.4)	4 (36.4)	1 (9.1)	4 (36.4)	8 (72.7)	5 (45.5)	0.58 (0.31)	62.45 (19.86)
Liver	12 (7.7）	4 (33.3)	0 (0)	2 (16.7)	9 (75.0)	3 (33.3)	0.67 (0.23)	64.75 (19.86)
Lung	49 (31.4)	17 (34.7)	8 (16.3)	17 (34.7)	34 (69.4)	18 (36.7)	0.58 (0.30)	56.37 (23.14)
Colorectal	47 (30.1)	15 (32.6)	5 (10.9)	13 (28.3)	26 (56.5)	16 (34.8)	0.62 (0.33)	58.70 (20.55)
*P* value		0.114	0.214	0.305	0.491	0.155	0.127	0.834
*Duration of cancer progression (days)*								
<Half years	66 (42.3)	20 (30.3)	11 (16.7)	20 (30.3)	39 (59.1)	27 (40.9)	0.59 (0.35)	56.23 (21.86)
Half to less than one year	35 (22.4)	14 (40.0)	3 (8.6)	11 (31.4)	23 (65.7)	14 (40.0)	0.59 (0.31)	60.97 (18.80)
Longer than one year	40 (25.6)	12 (30.0)	6 (15.0)	12 (30.0)	24 (60.0)	12 (30.0)	0.62 (0.29)	55.15 (25.27)
Missing	15 (9.6)							
*P* value		0.384	0.335	0.781	0.807	0.353	0.840	0.404
*Oncology therapies*								
No	63 (40.4)	21 (33.3)	9 (14.3)	17 (27.0)	44 (69.8)	22 (34.9)	0.60 (0.32)	58.83 (20.86)
Yes	91 (58.3)	27 (29.7)	11 (12.1)	28 (30.8)	48 (52.7)	34 (37.4)	0.62 (0.32)	57.74 (23.14)
Missing	2 (1.3)							
*P* value		0.394	0.303	0.893	0.106	0.839	0.566	0.981

NS: Not significant. ^*∗*^*p* < 0.05 duration of cancer progression (days): it is the duration between the time when filling out the questionnaires and the time of diagnosis of cancer progression. One-way ANOVA was applied if the homogeneity of variance assumption was satisfied, and the non-parametric Kruskal–Wallis H test was utilised if the homogeneity of variance assumption was not satisfied.

**Table 2:** Distribution of EQ-5D-5L dimension responses at three time points (*T*0, *T*1, and *T*2) and chi-square test results reporting the percentage of patients with moderate and severe problems across three time points.

Dimensions	*T*0 *N* (%)/*M* (SD)	*T*1 *N* (%)/*M* (SD)	*T*2 *N* (%)/*M* (SD)	Chi-square (*T*0, *T*1, *T*2) (*χ*^2^, *P*)/One-way ANOVA	Chi-square (*T*1, *T*2) (*χ*^2^, *P*)/One-way ANOVA	Chi-square (*T*0, *T*2) (*χ*^2^, *P*)/One-way ANOVA
*Mobility*						
No problems and slight problems (level ≤ 2)	74 (71.8)	69 (67.0)	77 (74.8)	*χ * ^2^ = 84.541 *p* < 0.001^^*∗∗*^^	*χ * ^2^ = 60.438 *p* < 0.001^∗∗^	*χ * ^2^ = 55.060 *p* < 0.001^∗∗^
Moderate, severe problems, and unable to do (level ≥ 3)	29 (28.2)	34 (33.0)	26 (25.2)			

*Self-care*						
No problems and slight problems (level ≤ 2)	92 (89.3)	88 (85.4)	85 (82.5)	*χ * ^2^ = 50.202 *p* < 0.001^∗∗^	*χ * ^2^ = 33.052 *p* < 0.001^∗∗^	*χ * ^2^ = 29.277 *p* < 0.001^∗∗^
Moderate, severe problems, and unable to do (level ≥ 3)	11 (10.7)	15 (14.6)	18 (17.5)			

*Usual activities*						
No problems and slight problems (level ≤ 2)	82 (79.6)	77 (74.8)	76 (73.8)	*χ * ^2^ = 78.562 *p* < 0.001^∗∗^	*χ * ^2^ = 53.007 *p* < 0.001^∗∗^	*χ * ^2^ = 48.228 *p* < 0.001^∗∗^
Moderate, severe problems, and unable to do (level ≥ 3)	21 (20.4)	26 (25.2)	27 (26.2)			

*Pain/Discomfort*						
No problems and slight problems (level ≤ 2)	46 (44.7)	55 (53.4)	63 (61.2)	*χ * ^2^ = 136.303 *p* < 0.001^∗∗^	*χ * ^2^ = 88.542 *p* < 0.001^∗∗^	*χ * ^2^ = 99.651 *p* < 0.001^∗∗^
Moderate, severe problems, and unable to do (level ≥ 3)	57 (55.3)	48 (46.6)	40 (38.8)			

*Anxiety/Depression*						
No problems and slight problems (level ≤ 2)	68 (66.0)	72 (69.9)	74 (71.8)	*χ * ^2^ = 91.625 *p* < 0.001^∗∗^	*χ * ^2^ = 60.027 *p* < 0.001^∗∗^	*χ * ^2^ = 64.254 *p* < 0.001^∗∗^
Moderate, severe problems, and unable to do (level ≥ 3)	35 (34.0)	31 (30.1)	29 (28.2)			

EQ-5D-5L index value	0.614 (0.318)	0.623 (0.330)	0.660 (0.323)	*F* = 0.093 *p* = 0.911	*F* = 0.155 *p* = 0.694	*F* = 0.114 *p* = 0.736
VAS	58.35 (22.205)	61.79 (21.207)	62.28 (22.832)	*F* = 1.145 *p* = 0.320	*F* = 0.027 *p* = 0.870	*F* = 1.813 *p* = 0.180

^*∗∗*^*p* < 0.01. Chi-square tests were used for categorical variables, while ANOVA tests were used for continuous variables.

**Table 3:** Results from the GEE model.

*Parameter estimates*										
Parameter	*B*	Std. Error	*95% Wald confidence interval*		*Hypothesis test*			Exp (*B*)	*95% Wald confidence interval for Exp (B)*	
			Lower	Upper	Wald chi-square	df	Sig.		Lower	Upper
(Intercept)	1.127	0.0653	1.000	1.255	298.209	1	0.000	3.088	2.717	3.509
ECOG-PS	−0.094	0.0138	−0.121	−0.067	46.754	1	0.000	0.910	0.886	0.935
Pain	−0.016	0.0047	−0.025	−0.007	11.630	1	0.001	0.984	0.975	0.993
Walking	−0.028	0.0078	−0.044	−0.013	13.185	1	0.000	0.972	0.957	0.987
HADS	−0.009	0.0015	−0.012	−0.006	34.462	1	0.000	0.991	0.988	0.994
(Scale)	0.033									

Dependent variable: EQ-5D-5L index score model: (Intercept), age range, disease duration range, sex, job, race, marital status, education, income, medical coverage, diagnosis, treatment, ECOG-PS, symptoms and interference (pain, fatigue, nausea, insomnia, distress, shortness of breath, memory, appetite, drowsy, dry mouth, sadness, vomiting, numbness, constipation, general activity, mood, work, relationship, walking, enjoyment), anxiety/depression from HADS, Time (baseline, *T*1-two week follow-up, *T*2-four week follow-up).

## Data Availability

All data supporting the findings of this study and all supplementary materials are available from the corresponding author upon reasonable request.
